# Carbamoyl-Phosphate Synthase 1 as a Novel Target of Phomoxanthone A, a Bioactive Fungal Metabolite

**DOI:** 10.3390/biom10060846

**Published:** 2020-06-02

**Authors:** Sara Ceccacci, Jana Deitersen, Matteo Mozzicafreddo, Elva Morretta, Peter Proksch, Sebastian Wesselborg, Björn Stork, Maria Chiara Monti

**Affiliations:** 1 Department of Pharmacy, Università di Salerno, Via Giovanni Paolo II 132, Fisciano, 84084 Salerno, Italy; sceccacci@unisa.it (S.C.); emorretta@unisa.it (E.M.); 2 PhD Program in Drug Discovery and Development, Department of Pharmacy, Università di Salerno, Via Giovanni Paolo II 132, Fisciano, 84084 Salerno, Italy; 3 Institute of Molecular Medicine I, Medical Faculty, Heinrich Heine University Düsseldorf, Universitätsstr. 1, 40225 Düsseldorf, Germany; jana.deitersen@uni-duesseldorf.de (J.D.); sebastian.wesselborg@uni-duesseldorf.de (S.W.); bjoern.stork@uni-duesseldorf.de (B.S.); 4 School of Biosciences and Veterinary Medicine, University of Camerino, Via Gentile III da Varano, 62032 Camerino, Italy; matteo.mozzicafreddo@unicam.it; 5 Institute of Pharmaceutical Biology and Biotechnology, Heinrich Heine University, Universitätsstr. 1, 40225 Düsseldorf, Germany; Peter.Proksch@hhu.de

**Keywords:** proteomics, drug affinity responsive target stability, targeted-limited proteolysis, bioactive xanthone, molecular docking

## Abstract

Phomoxanthone A, a bioactive xanthone dimer isolated from the endophytic fungus *Phomopsis* sp., is a mitochondrial toxin weakening cellular respiration and electron transport chain activity by a fast breakup of the mitochondrial assembly. Here, a multi-disciplinary strategy has been developed and applied for identifying phomoxanthone A target(s) to fully address its mechanism of action, based on drug affinity response target stability and targeted limited proteolysis. Both approaches point to the identification of carbamoyl-phosphate synthase 1 as a major phomoxanthone A target in mitochondria cell lysates, giving also detailed insights into the ligand/target interaction sites by molecular docking and assessing an interesting phomoxanthone A stimulating activity on carbamoyl-phosphate synthase 1. Thus, phomoxanthone A can be regarded as an inspiring molecule for the development of new leads in counteracting hyperammonemia states.

## 1. Introduction

**Figure 1:**
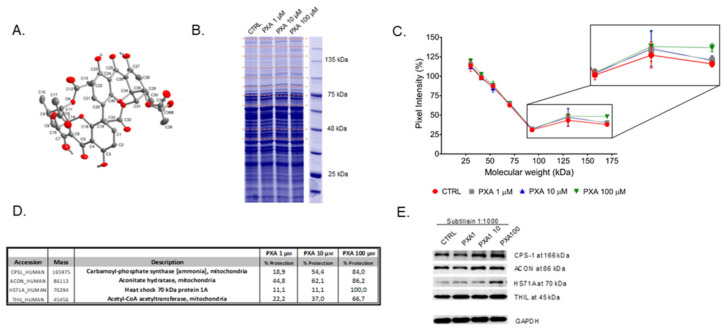
(**A**) Phomoxanthone A (PXA) structure. (**B**) Coomassie stained gel of samples treated without (CTRL) or with 1 µM, 10 µM or 100 µM of PXA and subtilisin at 1:1000 (w/w). Red lines indicate gel regions digested on one SDS-PAGE, as an example. (**C**) Densitometric analysis of the SDS-PAGE gels in Figure S1 reported, through GraphPad Prism, as the pixel intensity of each gel region vs. molecular weight. The major variation of pixel intensity of PXA-treated samples can be observed at MW higher than 75 kDa. (**D**) List of putative PXA interacting proteins together with the percentage of protection calculated as (matches of PXA treated sample – matches of control sample)/(matches of undigested lysate – matches of control sample)*100. (E) Immunoblotting analysis with opportune antibodies using subtilisin at 1:1000 (w/w). Glyceraldehyde 3-phosphate dehydrogenase (GAPDH) has been used as a loading normalizer.

Phomoxanthone A (PXA, Figure 1A, see below) is a xanthone dimer that is isolated from the endophytic fungus *Phomopsis* sp., which is known as a wealthy source of bioactive secondary metabolites endowed with a broad range activity against *Plasmodium falciparum* and *Mycobacterium tuberculosis* [1]. In particular, PXA shows a widespread bioactivity and, along the years, it has been tested on a large variety of cellular systems and pathways. Initially, PXA showed significant pro-apoptotic properties when tested on human cancer cell lines, whereas it was inactive on healthy normal cells. Interestingly, PXA was also an effective activator of natural killer cells, murine T lymphocytes, and macrophages, coding for a stimulation of the immune system together with its pro-apoptotic activity. This dual effect could help in counteracting resistance phenomena during chemotherapy [2,3]. More recently, PXA also displayed robust cytotoxicity against different solid tumor cells and their cisplatin-resistant sub-cells, with IC_50_ values in the high nanomolar/low micromolar range, as in ovarian cancer and bladder cancer cell pairs [4].

Besides, PXA has been considered a mitochondrial toxin, since its action selectively induces Ca^2+^ release from the mitochondria, but not from the endoplasmic reticulum, as reported by Böhler et al. [5]. This event depolarizes the mitochondria diminishing cellular respiration and electron transport chain activity, and it is accompanied by a rapid fragmentation (fission) of the mitochondrial network structure. This breakup solely includes the inward mitochondrial membrane, leading to cristae breakdown, the release of pro-apoptotic proteins, and apoptosis. Thus, mitochondrial ion homoeostasis and membrane dynamics seem to be affected by PXA [5]. On the basis of this broad biological profile and due to the limited information about the macromolecular targets involved in PXA action, a multi-disciplinary strategy has been developed and applied for identifying PXA target(s) to fully address its mechanism of action. In the early past, the mass spectrometry-based affinity purification approach (AP-MS) was one of the most recognized strategies for target identification of the so-called small molecules [6,7,8,9]: the indispensable requirement for fishing out protein interactors from a complex mixture is the covalent binding of the compound to a solid matrix to create the fishing bait. Thus, this method is limited to molecules standing with one or more functional groups with proper reacting outlines. Clearly, the compound under analysis has to be stable during the reaction with its matrix in the chosen solvent and, unfortunately, this is not the case for PXA. Indeed, PXA becomes unstable when dissolved in dimethyl sulfoxide, since it isomerizes into dicerandrol C [5]. To overcome these restrictions, many strategies have newly been developed for the target protein(s) detection of unmodified small molecules, such as DARTS (Drug Affinity Responsive Target Stability) [10,11,12,13]. DARTS is based on the ability of the tested compound to protect the interacting protein(s) against enzymatic proteolysis, as easily revealed by gel electrophoresis (SDS-PAGE). Later on, high-resolution MS can identify the targets by proteomics [12,13]. Furthermore, a more detailed depiction of the molecular mechanism of binding between a small molecule and its putative targets can be accomplished by t-LiP-MRM (targeted-Limited Proteolysis-Multiple Reaction Monitoring), which is a *gel-free* DARTS-like strategy based on a double-protease action and Multiple Reaction Monitoring-Mass Spectrometry (MRM-MS) detection. T-LiP-MRM points to the discovery of the local protein structural alterations due to complex formation with the small molecule [13,14,15]. Since PXA is unsuited for AP-MS analysis as reported above, DARTS and t-LIP-MRM were chosen to reveal its cellular targets in mitochondrial protein mixtures, which were isolated from HeLa cells. Mitochondria were selected as protein source since previously described PXA activities are related to this organelle. Next, the identified targets were validated by Western blotting analysis, in silico and in vitro biochemical experiments. More in detail, the gathered data led to the identification of carbamoyl-phosphate synthase 1 (CPS1) as a major PXA target, giving also detailed insights into the ligand/target interaction site and assessing an interesting PXA stimulating activity on CPS1. Thus, PXA and its analogues can be regarded as inspiring molecules for the development of new leads in counteracting hyperammonemia states.

## 2. Materials and Methods

### 2.1. Purification of Mitochondria from HeLa Cells

HeLa wild type cells (cells from Henrietta Lacks’s cancerous cervical tumor) were cultivated on 150 mm diameter tissue culture-treated dishes (Sarstedt) and approximately 3.6 × 10^8^ cells were harvested the next day via scraping. Cells were pelleted at 500× *g* for 5 min and washed twice with PBS (phosphate buffered saline, Gibco, Waltham, MA, USA). The pellet was re-suspended in 10 mL mitochondria isolation buffer (210 mM mannitol, 70 mM sucrose, 1 mM EDTA (ethylenediaminetetraacetic acid), 20 mM HEPES (4-(2-hydroxyethyl)-1-piperazineethanesulfonic acid) and protease inhibitor cocktail (Sigma-Aldrich, St. Louis, Missouri, USA, #P2714) for 5 min on ice before rupturing by seven strokes through a 26 G canule. Then, the cell lysate was centrifuged at 1000× *g* and 4 °C for 5 min, and the supernatant was collected. The remaining pellet of non-lysed cells was re-suspended in 2 mL of mitochondria isolation buffer and ruptured again before centrifugation, and the two fractions were pooled. The pooled lysate was centrifuged again at 1000× *g* and 4 °C for 5 min, and the pellet was discarded. The remaining lysate was centrifuged at 8000× *g* and 4 °C for 10 min. The supernatant (cytosolic fraction) was collected and centrifuged again before transferring into a new tube and freezing in liquid nitrogen. The pellet (mitochondrial fraction) was washed three times at 8000× *g* for 10 min in 250 µL mito isolation buffer. The pellet was finally centrifuged at 10,000× *g* and 4 °C for 10 min. The supernatant was discarded, and the pellet containing isolated mitochondria was frozen in liquid nitrogen and stored at −80 °C.

### 2.2. Identification of PXA Cellular Target(s) through DARTS

Mitochondria were lysed in a buffer composed of 1.5% digitonin, a protease inhibitor cocktail (Sigma Aldrich), 150mM of NaCl, 10mM of Tris/HCl (pH 7.5) and 5mM of EDTA. After 15 min at 4 °C, debris were removed by centrifugation at 20,000× *g* (30 min at 4 °C). Protein concentration of the obtained supernatant was determined by Bradford assay (BioRad Laboratoties, Hercules, CA) and subsequently adjusted at 3 mg/mL. DARTS experiments were carried out as reported by Morretta et al. [13]. Briefly, 300 μg proteins aliquots were either incubated with DMSO (vehicle control) or with PXA (1, 10, and 100 μM final concentrations) for 1h at room temperature and under agitation. Then, the obtained samples were treated with the unspecific protease subtilisin (enzyme to proteins ratio of 1:1000 w/w) and leaved shaking for 30 min at 25 °C. One aliquot of the DMSO-treated sample went a mock proteolysis to be kept as a reference. Then, the protease was quenched by adding PMSF (phenylmethylsulfonyl fluoride, Sigma-Aldrich, St. Louis, USA, 1 mM final concentration) to each sample. Subsequently, all of the samples were boiled in SDS-PAGE loading buffer (60 mM Tris/HCl pH 6.8, 2% SDS, 0.001% bromophenol blue, 10% glycerol, 2% 2-mercaptoethanol) and 20 μg were loaded on a 4%–12% Bis-Tris Criterion^TM^ XT Precast Gel (BioRad Laboratoties), which was then stained with a Comassie solution and submitted to a densitometric analysis through ImageJ. This experiment was carried out in duplicate. Thus, protein bands whose intensity raised at increasing PXA amounts were excised from the gels and submitted to an in situ tryptic digestion protocol [16]. Briefly, gel slices were reduced (6.5 mM 1,4-dithiothreitol, DTT), alkylated (54 mM iodoacetamide, IAA), washed and rehydrated, on ice for 1 h, in a 12 ng/µl trypsin/LysC solution (Promega, Madison, Wisconsin). Then, the enzymes excess was removed and replaced with ammonium bicarbonate (AmBic, 40 µl, 50 mM, pH 8.5), allowing protein digestion to proceed overnight at 37 °C. Subsequently, supernatants were collected and peptides were extracted from each gel slice, shrinking them in 100% CH_3_CN. The obtained peptides mixtures were dried under vacuum and dissolved in formic acid (FA, 10%) for the MS analysis.

Then, 5 µl of each sample were injected into a nano-ACQUITY UPLC system (Waters, Milford, MA, USA), separating peptides on a 1.7 µm BEH C18 column (Waters) at a flow rate of 280 nl/min. Peptide elution was achieved with a linear gradient of mobile phase B from 20% to 90% in 65 min (mobile phase A: 95% H_2_O, 5% CH_3_CN, 0.1% acetic acid; mobile phase B: 95% CH_3_CN, 5% H_2_O, 0.1% acetic acid). MS and MS/MS data were acquired on an LTQ Orbitrap XL high-performance liquid chromatography MS system (Thermo-Scientific, Waltham, MA, USA), provided with an electrospray (ESI) source. The 10 most intense doubly and triply charged peptide ions were fragmented. Then, MS data were processed by the MS Converter General User Interface software (ProteoWizard 3.0, Palo Alto, CA, USA; available online: http://proteowizard.sourceforge.net) to generate peak lists for protein identifications. Subsequently, database searches were carried out on Mascot Deamon (version 5.1, Matrix Science, London, UK), employing the SwissProt database (release November 2019, 561344 entries) and the following settings: two missed cleavages; carbamidomethyl (C) as fixed modification; oxidation (M) and phosphorylation (ST) as variable modifications; peptide tolerance 30 ppm; MS/MS tolerance 0.8 Da.

### 2.3. Western Blotting

First, 5 µg of samples from the duplicate experiments were analyzed by a 12% SDS-PAGE and transferred onto a nitrocellulose membrane; then, they were incubated for 1 h in a blocking solution (30 mM Tris pH 8, 170 mM NaCl, 3.35 mM KCl, 0.05% Tween-20, 5% non-fat dried milk) and left for 16 h at 4°C with monoclonal antibodies against CPS1, aconitate hydratase (ACON), heat shock 70 kDa protein 1A (HS71A), and acetyl-CoA acetyltransferase (THIL) (1:1000, Proteintech, Manchester, UK). Then, a mouse peroxidase-conjugated secondary antibody (1:2500; Thermo-Scientific) was added, and the signal was detected using an enhanced chemiluminescent substrate and LAS 4000 (GE Healthcare, Waukesha, WI, USA) digital imaging system. Finally, an antibody against glyceraldehyde 3-phosphate dehydrogenase (GAPDH) (1:2000 in 5% milk; Invitrogen, Carlsbad, CA, USA) has been tested as a loading normalizer.

### 2.4. T-LiP-MRM Analysis

CPS1 peptides previously detected by MS were selected through the proteomics data resource Peptide Atlas, available online: http://www.peptideatlas.org/ (accessed on 24/06/2019). They were subsequently queried in the complete human SRM Atlas, available online: https://db.systemsbiology.net/sbeams/cgi/PeptideAtlas/GetTransitions (accessed on 24/06/2019) to get a list consisting of 52 precursors, each one presenting the three most intense daughter ions. T-LiP-MRM experiments were carried out as reported by Morretta et al. [13].

### 2.5. In Silico Prediction of the PXA/CPS1 Complex

The molecular docking analysis between PXA and CPS1 was performed to predict the best complexes and it included the ligand and protein preparation, the genetic algorithm (GA) execution, the data analysis, and the final image preparation. PXA was designed, including the addition of tautomeric states, partial charges, and protonation, and finally minimized using the Avogadro software (version 1.2.0, Kitware, Clifton Park, New York, NY) [17] with a universal force field (UFF) and a conjugate gradient algorithm until a *ΔE* lower than 0.001 kJ/mol, as previously reported [18]. The universal force field (UFF) was developed to provide a set of rules and procedures for producing appropriate parameters across the entire periodic table. The CPS1 three-dimensional structure was obtained from the Protein Data Bank [19] (pdbID: 5DOU) [20] and prepared using the Hermes software (version 1.10.0, CCDC, Cambridge, UK) [21] incorporating the Gasteiger (Marsili) partial charges, adding polar protons, and removing crystal waters and extra co-crystallized ligands (see also Figure S3). Moreover, all planar R-NR1R2 were made available for the cis/trans flipping, and the tautomeric states of Asp, Glu, and His residues were adjusted. GOLD (version 5.7.0, CCDC, Cambridge, UK) [21,22] was performed to achieve the molecular docking using the ChemScore as scoring function, search efficiency at 200% (very flexible), selecting all atoms within 20Å from three centroids (namely the residues used as centroid were Leu134, Val647, and Leu778) to completely cover the whole protein, 20 GA runs, and other parameters as default. The resulting ChemScore *ΔG*, the total free energy change of the system upon ligand binding, and the relationship between this score and experimental free energy of binding, previously obtained, were used to calculate the predicted equilibrium dissociation constant K_D_. The best complex geometry, on the base of the ChemScore and the ChemScore *ΔG*, was rendered using PyMol software (The PyMOL Molecular Graphics System, Version 2.3.4 Schrödinger, LLC, New York, NY), whereas the 2D representation was created using the PoseView server [23].

### 2.6. CPS1 In Vitro Activity Assay

The reaction mixture consisted of 50 mM NH_4_HCO_3_, 10 mM Mg(C_2_O_2_H_3_)_2_, 5 mM ATP, 5mM N-acetyl-L-glutamate (NAG), 1 mM dithiothreitol, 50 mM triethanolamine, 50 µg/mL of CPS1 in a final volume of 20 µL. The reaction was run for 10 min at 37 °C, and the resulting carbamoyl phosphate was converted to hydroxyurea by the addition of 100 mM hydroxylamine and incubation for 10 min at 95 °C. To measure the concentration of hydroxyurea, 80 µL of chromogenic reagent was added to the reaction tubes followed by heating for 15 min at 95 °C. The chromogenic reagent consisted of equal volumes of solutions A and B, which were mixed immediately before use. Solution A consisted of 8 mg of antipyrine dissolved in 1 mL of 40% (v/v) H_2_S0_4_. Solution B contained 6 mg of diacetyl monoxime in 1 mL of 5% (v/v) acetic acid. After cooling to room temperature, 30 µl were loaded in triplicate in a 384-multiwell plate, and the absorbance was measured at 458 nm on a MultiskanGO Spectrophotometer (Thermo-Scientific, Waltham, MA, USA). Opportune controls were performed such as the measure of PXA absorbance at the different used concentrations. The same experiment has been carried out using Serratol (also diluted in DMSO), a component of the frankincense essential oil, as negative control. Indeed, Serratol did not affect CPS1 activity at tested concentrations from 100 to 500 µM (Figure S2).

## 3. Results and Discussion

Due to its intrinsic instability, PXA has not been considered appropriate for AP-MS strategy and the subsequent covalent modification. For this reason, both DARTS and t-LiP-MRM approaches were applied and improved for the detection of unmodified PXA targets in an unbiased way: DARTS-MS has been used to identify the main targets of the molecule, confirmed or not by Western blotting analysis. Then, t-LiP-MRM and molecular docking have been performed to shed light on the molecule putative interaction site(s). Finally, in vitro assays to evaluate PXA biological properties were performed.

### 3.1. Identification of PXA Cellular Target(s) through DARTS

In a pseudo-physiological environment, a protein is in dynamic equilibrium with multiple conformations but, upon ligand binding, the equilibrium will move to the bound state stabilized by possible hydrogen bonding, hydrophobic, and/or electrostatic interactions. This drives to a thermodynamically more stable state in which the protein breathing is lowered and the target resistance to proteolysis is definitely enlarged [24,25].

Thus, the limited proteolysis of a total (not fractionated) native cellular lysate, in the presence or in absence of PXA, has been carried out using an unspecific enzyme as subtilisin, and the digestion profile has been compared by SDS-PAGE: in principle, the bands of the protein targets should be more colored in the samples pre-treated with PXA compared to the control ones, and they can be identified through classical proteomic approaches [16]. Here, non-denatured mitochondrial lysates from HeLa cells were incubated with PXA for 1 h (from 1 to 100 µM) and then treated with subtilisin in controlled conditions of pH, time, and temperature. The samples were resolved by SDS-PAGE and revealed by Coomassie (Figure 1B and Figure S1): following the densitometric analysis in Figure 1C, those bands whose intensity enhanced in the lanes containing increasing concentration of PXA were cut (see red dotted lines, Figure 1B) and digested as reported by Shevchenko [16]. The entire experimental flow chart has been repeated twice, and all of the samples were analyzed through nano-ultra performance liquid chromatography coupled to high-resolution tandem mass spectrometry (nano-UPLC-MS/MS), followed by a Mascot database search, to obtain protein identification. In order to estimate, in a semi-quantitative way, the PXA protection levels calculating a protection percentage, Mascot protein matches outputs (Table S1) were compared for all identified proteins at their own molecular weight. The proteins whose protection was clearly dependent on PXA were comprised in the list of its putative interacting partners (Figure 1D); namely they are acetyl-CoA acetyltransferase (THIL), aconitate hydratase (ACON), carbamoyl-phosphate synthase-1 (CPS1), and heat shock 70 kDa protein 1A (HSP71A). The direct contact of PXA with its putative targets, and accordingly their low susceptibility from subtilisin, was then verified by Western blotting analysis, carried out with appropriate antibodies to have a better quantitative measurement of the PXA protection grade, independent by mass spectrometry (Figure 1E). Indeed, the comparison of PXA-treated samples showed increasing intensities (from left to right) of both CPS1 and HSP71A corresponding bands (MW of 160 and 70 kDa, respectively), while a significant variation of signal intensity was evident neither for ACON nor for THIL. A densitometric analysis has been carried out, using GAPDH (glyceraldehyde 3-phosphate dehydrogenase) as loading control (Figure S2). Since HSP71A is principally localized in the cellular nucleus and cytoplasm, the characterization of PXA binding to the mitochondrial enzyme CPS1 has been carried out.

### 3.2. Analysis of PXA/Targets Interaction Area by t-LiP-MRM

DARTS allows identifying the putative interactome of PXA; following, t-LiP-MRM has been applied on CPS1 in order to go deep into the interaction features. Indeed, t-LiP-MRM allows the identification of the target/ligand interaction peptides due to the protein structural changes induced by the small molecule, without any purification of the protein itself in a whole cell lysate.

This protocol is based on a double-protease digestion procedure: in a first step, the proteolysis by an unspecific enzyme is carried out in limited conditions on native samples; afterwards, the sample are denatured and fully hydrolysed by trypsin. Thus, a mixture of semi-tryptic and fully tryptic peptides is produced; the latter is suitable for targeted MRM-MS and quantification analysis. Definitely, fully tryptic peptides abundances are related to the target local conformational changes due to ligand binding. More in the deep, an in silico search using the bio-informatics tool Peptide Atlas allows to write the MRM methods, setting the best MRM transitions of the theoretical CPS1 fully tryptic peptides to map the protein sequences.

Next, mitochondria lysate samples were incubated with 1 and 10 µM PXA or vehicle (negative control) and treated with subtilisin at 1:1500 w:w enzyme to proteins ratio, under non-denaturing conditions. Then, samples were denatured and submitted to a complete tryptic digestion, giving peptides mixtures suitable for UPLC-MRM-MS analysis. A comparison of intensities of the fully tryptic peptides of PXA-treated and untreated runs revealed the interaction regions between PXA and CPS1, namely the peptides protected from subtilisin action. Peptides whose intensity raised up in the samples exposed to the molecule were selected as symptomatic of protection on specific CPS1 regions (Table 1). 

**Figure 2:**
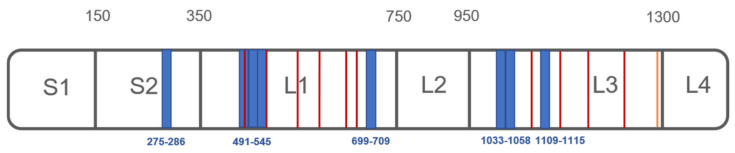
Schematic CPS1 cartoon covering different domains is depicted in gray, with the LiP-protected peptides highlighted in blue, ADP binding sites residues in red (T502, E503, R505, M543, R545, E581, K582, V584, E589, M614, H617, Q658, E672, N674, R676 and R679, R1087, K1126, V1128, E1133, H1160, Q1201, E1213, R1217, R1220), and T’-loop in orange (P1269-G1291).

In particular, peptides mapping for CPS1 interaction sites were identified around different regions: peptides G-[275-286]-K, V-[491-505]-R, I-[519-533]-K, A-[534-545]-R, I-[699-709]-K, I-[1033-1043]-R, S-[1048-1058]-K, and F-[1109-1115]-R resulted to be protected by both concentrations of PXA in three experiments. The first peptide resides in the so-called S2 region, and the following four peptides belong to the active bicarbonate phosphorylation site (L1 region, Figure 2). Moreover, the latter three peptides belong to the so-called carbamate phosphorylation site, which is the other CPS1 catalytic site (L3 region, Figure 2). Thus, even though no data about the stoichiometry of the complex between CPS1 and PXA were obtained, it seems that PXA can interact on the protein surface within different sites, promoting the protection of several peptides in L1, L3, and S2 (Figure 2).

### 3.3. Molecular Docking Analysis of PXA/CPS1 Complex

**Figure 3:**
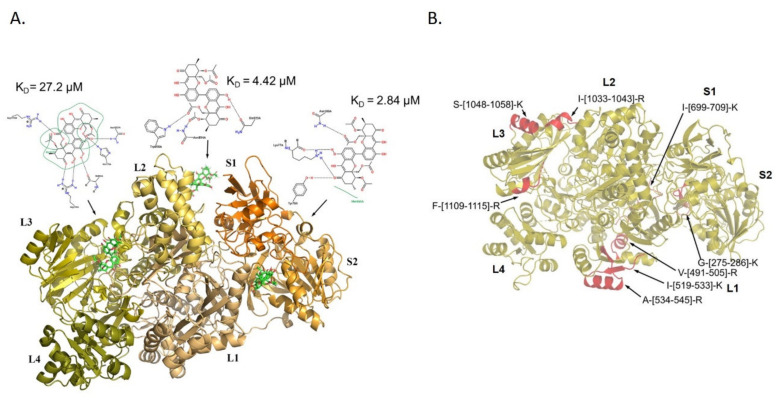
(**A**) The three best predicted complexes between PXA and CPS1 obtained using the molecular docking. CPS1 regions, showed in cartoon mode, are labeled and reported with different colors. Equilibrium dissociation constants K*_D_* for each binding pose and the amino acids involved in the interaction with PXA (showed as green sticks) are reported in the 2D pose depictions. (**B**) CPS1 3D structure is depicted in gold and the PXA-protected peptides identified by targeted-Limited Proteolysis-Multiple Reaction Monitoring (t-LiP-MRM) in red and marked with the corresponding identifiers.

In parallel, a molecular docking analysis of PXA on the CPS1 protein has been performed using the 3D structure of the human protein with a resolved crystallographic structure (pdb ID 5DOU) [17,18,19,20]. On the basis of the predicted affinity, PXA shows the best interaction poses into three different protein binding sites: in particular, as reported in Figure 3A, PXA interacts with the aminoacids R738, H775, I944, N1021, and R1195, into the interspace between the L2 and L3 region, with a K_D_ of 27.2 µM; with the amino acids N894, W898, and Q923, in the L2 region, with a K_D_ of 4.42 µM; and with the amino acids K77, Y78, N188, and M666 into the interspace between S2 and L1 region, with a K_D_ of 2.84 µM. Comparing the t-LIP-MRM data with those obtained by molecular docking, it seems clear that the L3 region of CPS1 undergoes a conformational variation, which is identified as a protection from proteolysis by t-LIP-MRM, namely the peptides I-[1033-1043]-R, S-[1048-1058]-K, and F-[1109-1115]-R, which is due to a direct interaction within PXA, as inferred by molecular docking (Figure 3B). In addition, the L1 region seems to be sheltered from proteolysis by t-LIP-MRM even if, accordingly to the molecular docking data, PXA does not lie in close proximity of this region but, possibly, inducing a middle term conformational variation (Figure 3B). On the other side, no experimental t-LIP-MRM evidences corroborate the molecular docking-based interaction of PXA in between L2 and S1 cavity. In Figure 2, the amino acids of two active sites of the protein (in L1 and L3 domains) are reported in red: those of the L1 domain involved in the binding of the nucleotide and phosphate are T502, E503, R505, M543, R545, E581, K582, V584, E589, M614, H617, Q658, E672, N674, R676, and R679, while those of the L3 domain are R1087, K1126, V1128, E1133, H1160, Q1201, E1213, R1217, and R1220. Looking at the putative PXA interaction sites disclosed by docking analysis or t-LIP-MRM (Figure 3A,B), it seems that two of them (in proximity of L3 and L2) are allosteric binding sites, whereas the PXA binding site reported in the L1 domain (K_D_ of 2.84 uM) can be considered orthosteric, since the residues T502, E503, R505, M543, and R545 of the active sites are in two peptides protected in t-LIP experiments. [20].

### 3.4. CPS-1 In Vitro Activity Assay

**Figure 4:**
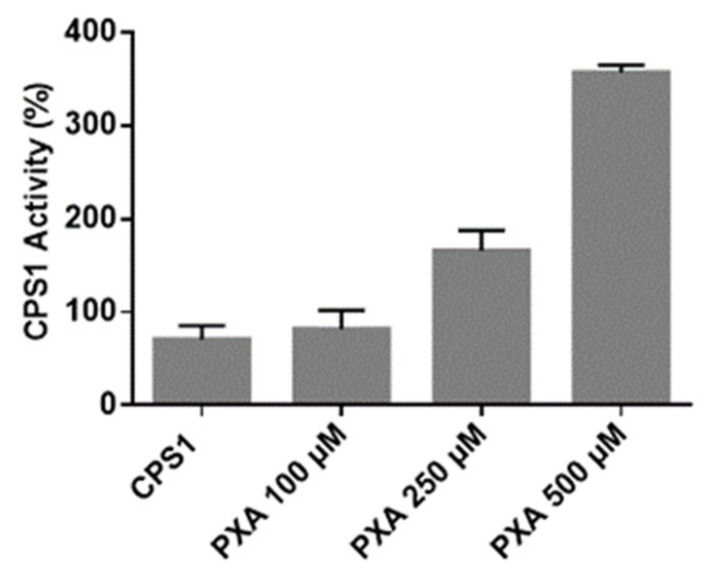
CPS1 activity measured as reported above in presence of different PXA concentrations. The data are shown as a percentage of CPS1 activity. The experiments have been repeated three times, and S.D. has been calculated. Opportune controls were carried out as the measurement of the signal of PXA alone at different concentrations (see also Figure S4).

Finally, in vitro activity assays were performed to monitor the effect of PXA on CPS1 activity. Human CPS1 is a 1462 amino acid, 160-kDa multi-domain mitochondrial, liver, and intestinal enzyme. It catalyzes the biosynthesis of carbamoyl phosphate (the first reaction of the urea cycle) in a multi-component reaction (2ATP + NH_3_ + HCO_3_^−^ → 2ADP + Pi + carbamoyl phosphate), which takes place in these sequential steps: bicarbonate phosphorylation, the synthesis of carbamate, and its phosphorylation. CPS1 is inactive in its apo-form and requires N-acetyl-L-glutamate (NAG) to reach an active conformation [26]. Its activity is correlated with many cellular functions and its dysfunction or deficit leads to many pathological conditions. In mammals, surplus nitrogen, mainly from protein catabolism, is mainly detoxified in the urea cycle: this pathway is responsible for ammonia detoxification and for endogenous arginine synthesis [26]. On the other side, CPS1 deficiency (CPS1D) generates urea cycle disorders (UCDs), a set of inborn diseases linked to nitrogen detoxification, which can undergo hyperammonemia [27,28,29]. Unless promptly treated, it can generate encephalopathy, coma, and death. The current treatment of CPS1D includes the use of N-carbamyl-L-glutamate, the commercial analogue of NAG, improving in vitro and in vivo the function of the enzyme. In order to test the effect of PXA on CPS-1 activity, an in vitro colorimetric assay has been developed. The reaction mixture containing CPS1, ATP, and NAG was run for 10 min at 37 °C [30] in the presence and absence of PXA at concentrations from 7.5 to 375 µg/mL. The resulting carbamyl phosphate was converted to hydroxyurea by the addition of hydroxylamine, and the reaction was stopped by heating the samples. To evaluate the hydroxyurea concentration, a chromogenic reagent was added, and the mixtures were loaded, in triplicate, in a 384-multiwell plate to measure the absorbance at 458 nm. As clearly shown in Figure 4, high PXA concentrations are able to significantly increase CPS1 enzymatic activity up to threefold. Since the peptide F-[1109-1115]-R, whose protease susceptibility has been altered by PXA, is in spatial proximity to the so-called T’-loop, which undergoes to a conformational variation upon NAG binding, ‘opening’ a tunnel channel for carbamate translocation, [20] it may be speculated that PXA favors this opening acting positively on CPS1 activity.

## 4. Conclusions

PXA and other phomoxanthones are secondary metabolites typical for the fungal genus *Phomopsis* [2]. Chemically, PXA is a homodimer of two tetrahydroxanthones with the two units covalently linked to each other via a biaryl bond with four hydroxy groups replaced by acetyl groups and four free alcoholic (phenolic) functions. The site of the bond between the two dimer subunits is the only structural difference between PXA and its less toxic isomers phomoxanthone B and dicerandrol C: in PXA, the two xanthonoid monomers are symmetrically linked at the position C-4,4′. Notably, PXA is unstable in polar solvents for a medium time with the covalent bond between the two monomers shifting between 2,2′-, 2,4′-, and 4,4′-linkage [31,32]. It is interesting that PXA was first identified as an antimalarial compound, revealing its strong antibiotic activity against the protozoan parasite *Plasmodium falciparum*, then against *Mycobacterium tuberculosis* and against two human cancer cells lines [1] Furthermore, its reported antibiotic activity has been investigated against the alga *Chlorella fusca*, the fungus *Ustilago violacea*, and the bacterium *Bacillus megaterium*. This broad range of activity against bacteria, protozoans, fungi, plants, and animal cells banned it as a specific antibiotic, promoting, on the other side, its anti-cancer profile, since it was more toxic against human cancer cells than non-cancer cells [2,3,4,5]. More recently, it has been enlightened that PXA might have an application as a tool in the study of mitochondrial membrane dynamics, particularly non-canonical mitochondrial fission and remodeling of the mitochondrial matrix. Indeed, PXA mainly causes cristae disruption and fragmentation of the matrix [3,4,5]. Intrigued by its spread biological profile, our MS-based proteomics research moved to the target(s) discovery of this natural compound through a combined strategy consisting of MS-based DARTS and t-LiP-MRM. All the gathered proteomics data, together with molecular docking analysis and biochemical in vitro assays, pointed to the unexpected discovery of CPS1 as one of the most reliable PXA partners, together with mitochondrial aconitate hydratase, heat shock 70 kDa protein 1A, and mitochondrial acetyl-CoA acetyltransferase. We pointed out our main interest on CPS1, since this mitochondrial enzyme acts a decisive task in getting rid of ammonia in excess into cells (mainly in intestinal epithelial cells and hepatocytes) by producing carbamoyl phosphate from ammonium, increasing arginine content and the substrate of pyrimidine synthesis in the so-called urea cycle. It is also expressed in several types of cancer cells including liver, colorectal, stomach, cervical, and pancreatic cancer cell lines [20]. On the other side, CPS1 deficiency is a rare autosomal genetics-based disease belonging to urea cycle disorders (UCD), producing a reduced metabolism of proteins and nitrogen and elevated ammonia levels in the cell. Ammonia is extremely toxic, especially for the nervous system, and high NH_3_ levels can result in retardation and convulsions. Moreover, hyperammonemia can happen after birth causing vomiting, hypothermia, seizures, and coma. The activation of CPS1 as detoxifying by NAG or its analogues is actually employed as pharmacological strategy, since they improved in vitro and in vivo the function of CPS1 and mutated isoforms [33]. It is very interesting that low CPS1 expression and hyperammonemia have been measured in Non-Alcoholic Steato Hepatitis (NASH) and Non-Alcoholic Fatty Liver Disease (NAFLD), which are very common diseases worldwide. Indeed, CPS1 can be considered as a potential novel target for the prevention of progression of both diseases: mainly, when the liver is affected, the enzymes converting nitrogen into urea are inhibited, leading to the growth of toxic ammonia that determines the scratch of tissue development, increasing the risk of disease progression [34,35]. Moreover, it has been reported that chemotherapy might induce hyperammonemia by CPS1 deficiency and, consequently, there is the need for urgent therapeutic strategies addressing a possible secondary urea cycle failure in patients affected by hyperammonemia during chemotherapy and stem cell transplantation [29]. Actually, a natural compound called fisetin, a plant polyphenol from the flavonoid group, has been tested in the treatment of induced hyperammonemic rats showing an increase of expression of several enzymes as CPS1 and a decrease of iNOS and NF-κB p65. It was observed that fisetin normalized ammonia levels, transaminases, and alkaline phosphatase in circulation, as well as glutamate and glutamine in the brain, and it also stabilized the circadian locomotor rhythm [36]. On the basis of this study, PXA could be considered an interesting probe for further investigations on the development of a new class of CPS1 positive modulators playing a pharmacological role in the therapy of several human diseases and metabolic disorders.

## Figures and Tables

**Table 1:** Selected carbamoyl-phosphate synthase-1 (CPS1) peptides reported with their Q1 m/z value, their length, the retention time in UPLC-MS, and the calculated fold changes. The fold change represents the ratio between the area of the tryptic peptide in the PXA-treated sample and the area of the tryptic peptide in the untreated sample. The same experiments have been repeated three times, and the fold changes were calculated over the means of the peptides’ area. *p* have been calculated, and only tryptic peptides with a *p* < 0.05 are reported.

Q1_m/z	Peptide	rt	Fold Change at PXA 1 µM	*p*	Fold Change at PXA 10 µM	*p*
663.36	G-[275-286]-K	6,7 min	1.73	0.0001	1.74	0.0003
804.4	V-[491-505]-R	9,58 min	1.77	0.0013	1.84	0.0008
795.43	I-[519-533]-K	12,45 min	2.57	0.0338	2.40	0.0473
653.85	A-[534-545]-R	9,13 min	1.30	0.0016	1.25	0.0033
582.37	I-[699-709]-K	11,24 min	1.23	0.0179	1.20	0.0422
615.83	I-[1033-1043]-R	8,04 min	1.44	0.0011	1.41	0.0013
611.34	S-[1048-1058]-K	13,44 min	1.88	0.0001	1.85	0.0001
433.22	F-[1109-1115]-R	5,05 min	1.34	0.0235	1.28	0.0315

## Supplementary Materials

The following are available online at https://www.mdpi.com/2218-273X/10/6/846/s1, Table S1: All proteins identified in two independent DARTS experiments together with the mean of their Mascot scores and matches; Figure S1: DARTS replicate experiments exploited for the densitometric analysis reported in Figure 1C. The left panel of this figure is reported in the manuscript as Figure 1B; Figure S2: Densitometric analysis of immunoblotting reported in Figure 1E was performed through ImageJ, and the histograms are the results of the quantitation of the signals from two independent experiments (standard deviations are reported). The undigested protein was rated as 100% in each analysis and GAPDH was exploited as a loading normalizer; Figure S3: ADP has been chosen as a known CPS1 ligand. The docking analysis of ADP on CPS1 has been carried out using the identical experimental parameters employed in PXA one. The superimposition is now reported in Figure S3, in which the light blue cartoon ADP from 5dou is comparable with the green cartoon of ADP conformation obtained by us. Root-mean-square deviation (RMSD) has also been reported; Figure S4: CPS1 activity measured by the reported assay in presence of another natural compound (Serratol) as control.
