# Supplementary material for: Carbamoyl-Phosphate Synthase 1 as a Novel Target of Phomoxanthone A, a Bioactive Fungal Metabolite
Source: Biomolecules. 2020 Jun 2;10(6):846. doi: 10.3390/biom10060846 (PMC7356042; doi:10.3390/biom10060846)
Supplement: Supplementary file 1 [file biomolecules-10-00846-s001.pdf]

biomolecules

Supporting information

Carbamoyl-phosphate synthase 1 as a novel target of phomoxanthone A, a bioactive fungal metabolite.

Sara Ceccacci [1,2], Jana Deitersen [3], Matteo Mozzicafreddo [4], Elva Morretta [1], Peter Proksch [5], Sebastian Wesselborg [3], Björn Stork [3] and Maria Chiara Monti [1,*]

[1] Dept. of Pharmacy, Università di Salerno, Via Giovanni Paolo II 132, 84084 Fisciano, Salerno, Italy; sceccacci@unisa.it (S.C.); emorretta@unisa.it (E.M.); mcmonti@unisa.it (M.C.M.).
[2] PhD Program in Drug Discovery and Development, Dept. of Pharmacy, Università di Salerno, Via Giovanni Paolo II 132, 84084 Fisciano, Salerno, Italy; sceccacci@unisa.it.
[3] Institute of Molecular Medicine I, Medical Faculty, Heinrich Heine University Düsseldorf, Universitätsstr. 1, 40225 Düsseldorf, Germany; jana.deitersen@uni-duesseldorf.de (J.D.); sebastian.wesselborg@uni-duesseldorf.de (S.W.); bjoern.stork@uni-duesseldorf.de (B.S.).
[4] School of Biosciences and Veterinary Medicine, University of Camerino, Via Gentile III da Varano, 62032 Camerino, Italy; matteo.mozzicafreddo@unicam.it (M.M).
[5] Institute of Pharmaceutical Biology and Biotechnology, Heinrich Heine University, Universitätsstr. 1, 40225 Düsseldorf, Germany; Peter.Proksch@hhu.de (P.P.).
* Correspondence: mcmonti@unisa.it.

Table S1. All proteins identified in two independent DARTS experiments together with the mean of their Mascot scores and matches. The ones in light gray are those for which both scores and matches increase when PXA is pre-incubated with the lysate in a concentration dependent mode.

Accession	Description	CTRL		PXA 1uM		PXA 10uM		PXA 100uM		Lysate	
		Score	Matches	Score	Matches	Score	Matches	Score	Matches	Score	Matches
THIL_HUMAN	Acetyl-CoA acetyltransferase, mitochondrial	1752	46	1786.5	49	2113	51	2174	55	2168	59.5
ACON_HUMAN	Aconitate hydratase, mitochondrial	1200	25	1619	38	1687	43	2379	50	2488.5	54
CALX_HUMAN	Calnexin	49	1	78	2	150	3	250	5	773	29.5
CPSM_HUMAN	Carbamoyl-phosphate synthase, mitochondrial	1758	55.5	2450.5	71.5	3507.5	101.5	4332.5	126.5	4391	140
ECI2_HUMAN	Enoyl-CoA delta isomerase 2, mitochondrial	663	16	1090	25	310	11	325	10	265	6.5
HS71A_HUMAN	Heat shock 70 kDa protein 1A	1090	25	1040	26	1061	26	1143	34	1161.5	34
PREP_HUMAN	Presequence protease, mitochondrial	199	8	293	15	360	13	236	14	176.5	8
PDIA4_HUMAN	Protein disulfide-isomerase A4	120	3	184	6	154	6	152	3	106	2.5
ROCK2_HUMAN	Rho-associated protein kinase 2	114.5	7	152	8.5	223.5	9.5	255.5	12	181.5	9
SYVC_HUMAN	Valine--tRNA ligase	151	6	72	4	139	4	80	4	71.5	4.3

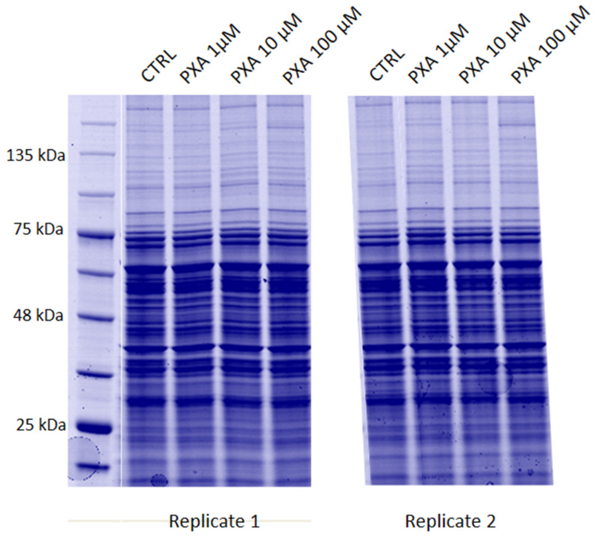

Figure S1. DARTS replicate experiments exploited for the densitometric analysis reported in Figure 1C. The left panel of this figure is reported in the manuscript as Figure 1B.

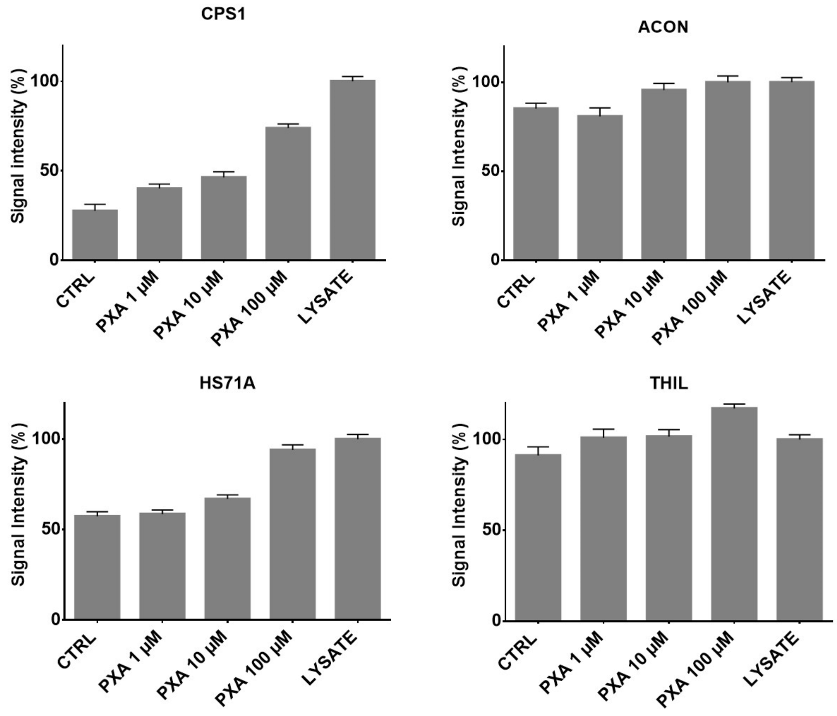

Figure S2. Densitometric analysis of immunoblotting reported in Figure 1E was performed through ImageJ and the histograms are the results of the quantitation of the signals from two independent experiments (standard deviations are reported). The undigested protein was rated as 100% in each analysis and GAPDH was exploited as a loading normalizer.

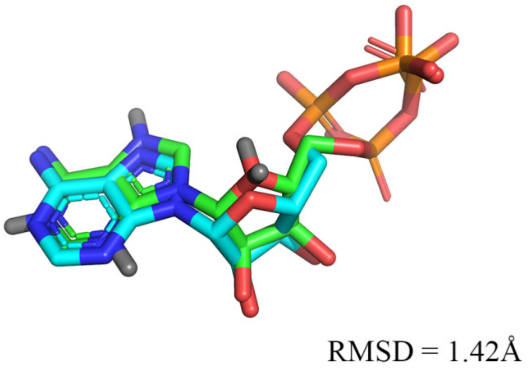

Figure S3. The docking analysis of ADP on CPS1 carried out using the identical experimental parameters employed in PXA one. The superimposition here reported shows the light blue stick ADP from 5dou in comparison with the green stick of ADP conformation obtained by docking analysis. Root-mean-square deviation (RMSD) has also been reported.

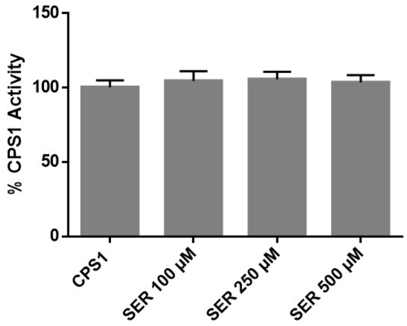

Figure S4. CPS1 activity measured by the reported assay in presence of another natural compound (Serratol) as control.
